# The role of consumer choice in out-of-pocket spending on health

**DOI:** 10.1186/s12939-023-01838-1

**Published:** 2023-01-31

**Authors:** Laura Nübler, Reinhard Busse, Martin Siegel

**Affiliations:** 1 Department of Empirical Health Economics, Technische Universität Berlin, H51, Straße des 17. Juni 135, 10623 Berlin, Germany; 2 Department of Healthcare Management, Technische Universität Berlin, Berlin, Germany; 3 Berlin Centre of Health Economics Research (BerlinHECOR), Berlin, Germany

**Keywords:** Out-of-pocket spending on health, Kakwani index, Consumer choice, Policy analysis, Decomposition analysis, Health insurance, Germany

## Abstract

**Background:**

Analyses of out-of-pocket healthcare spending often suffer from an inability to distinguish necessary from optional spending in the data without making further assumptions. We propose a two-dimensional rating of the spending categories often available in household budget survey data where we consider the requirement to pay for necessary healthcare as one dimension and the incentive to pay extra for additional services, higher quality options or more convenience as a second dimension to assess the distortionary potential of higher spending for additional healthcare or higher quality options.

**Methods:**

We use three waves of a large German Household Budget Survey and decompose the Kakwani-index of total out-of-pocket healthcare spending into contributions of the eleven spending categories available in our data, across which user charge regulations vary considerably. We compute and decompose Kakwani-indexes for the different spending categories to compare the degrees of regressiveness across them.

**Results:**

The results suggest that categories with higher incentives for additional spending exhibit smaller contributions to the overall regressive effect of total out-of-pocket spending than categories where spending is presumably mostly on necessary and effective care.

**Conclusions:**

Assessing the consumer choice potential of different spending categories is important because extra spending among the better-off may outweigh necessary spending in aggregate expenditure data, and may also hint at potential inequalities in the quality of provided healthcare.

## Introduction

Higher out-of-pocket (OOP) spending on elective and luxury options among richer households may mask over-proportional spending on basic medical goods and treatments among the poor. However, budget surveys rarely include information on need, and data on OOP spending on healthcare usually allow no distinction between higher spending on more intensive utilization of necessary healthcare and higher spending on pricier options or additional services without further assumptions. This paper proposes a policy analysis based approach to identify healthcare spending categories that are particularly susceptible to consumer choice biases, and compares the distributions of OOP spending in the different categories. The aim is to contribute to a better understanding of the role of consumer choices in OOP spending on healthcare.

Effects of healthcare funding schemes on income are typically assessed by Kakwani’s inequality-based measure of tax proportionality [1–10]. The Kakwani index considers a financing scheme progressive if larger income shares are collected from richer than from poorer households, and regressive if the poor are over-proportionally charged. In proportional schemes, all households contribute the same fraction of their incomes. Recent applications of the Kakwani index report significantly more progressive distributions of OOP spending on healthcare in categories with different price and quality options than in categories with a limited range of options [11–14], and some argue that consumer choices and additional spending by richer households may have distorted these estimates [13, 14]. Given that medical needs are usually concentrated towards the poor [15–22], and that user charges are usually not determined according to income, progressivity does not necessarily indicate a fair distribution of OOP spending on medical necessities, but may instead result from richer households’ choices to afford additional or higher quality healthcare. Treating all medical expenditures equally without distinguishing basic spending from elective and luxury spending may therefore result in a consumer choice bias: The Kakwani index may indicate more progressivity (or less regressivity) than it would if only expenditures for basic healthcare were included.

This paper proposes to first analyze user charge regulations to rate categories in two dimensions: by the requirement to pay for basic, necessary care and by the incentive to pay extra for additional services, better quality options or convenience. We use the co-payment scheme of the German Statutory Health Insurance (SHI) as an example because the regulations for user charges and the range of available options vary considerably across different spending categories. For example, all ambulatory healthcare considered necessary and effective is covered by the SHI and requires no co-payments, whereas for dentures, a fixed sum is reimbursed and any excess cost is covered by the patient. Furthermore, using a large German household budget survey (*Einkommens- und Verbrauchsstichprobe, EVS* [23–25]) allows us to distinguish healthcare spending in eleven different categories. We then compare the degree of regressivity across the different healthcare spending categories using the Kakwani index and investigate how regressivity varies across categories with different requirements to pay OOP for basic healthcare, and with different incentives to pay OOP for additional healthcare.

## Institutional background and classification of spending categories

### Institutional background

The Social Code Book V (SGB V) provides the legal framework for the German SHI. Approximately 87% of the German population are covered by the SHI, of which the majority are compulsory members. Civil servants, high gross income earners (exceeding 4,950 Euro in 2018) and self-employed individuals are exempt from compulsory SHI but may become voluntary members [26, 27]. Coverage includes prescribed medicines and anything from simple ambulatory consultations to screenings and preventive treatments, expensive medicines and complex procedures in ambulatory, inpatient and dental care, if deemed necessary and effective. The regulations for SHI use a rather generous notion of necessary and effective care, which not only includes emergency treatments in life-threatening situations, but any acknowledged treatment or medicine to maintain or improve a patient’s health and to prevent the onset of diseases. All sickness funds must at least cover a certain benefit package [26–28].

The 11 OOP payment categories adopted from the EVS data allow choices to different degrees. For example, all necessary and effective ambulatory medical services are covered free of charge. However, ambulatory care providers may offer additional services (*IGeL-Leistungen*) which cannot be reimbursed. The admission fee (*Praxisgebühr*) was charged for the first ambulatory healthcare utilization in a quarter until 2012 regardless of health status or healthcare provided and is only relevant for the 2008 survey. Medical equipment and aids include glasses, wheelchairs and other potentially expensive items, but only basic options are reimbursed. Several options exist for crowns and dentures, but reimbursements for dentures and materials are fixed at 50-65% of the average cost for basic options. Covered dental care services are free of charge, but dentists may offer additional services (*IGeL-Leistungen*) which cannot be reimbursed. Inpatient care is subject to daily co-payments, and hospitals may charge extra for extra services and better accommodation. Prescribed medicines are subject to a co-payment of 5-10 Euros, but medicines and medical goods without prescription are not covered and must be fully paid by the patient. See [26–28] for more details on the German SHI.

### Assessment of the consumer choice bias potential

We rate the different spending categories in two dimensions with respect to their different regulations. The first dimension addresses the user charges for standard healthcare (*Regelversorgung*). We rely on the German SGB V, which demands that all necessary and effective healthcare must be covered by the SHI, and distinguish covered (presumably the necessary and effective minimum) from additional healthcare in our analysis.

We consider the *requirement to pay OOP for covered care* as low if low OOP payment is required (e.g. for ambulatory care or prescribed medicines), and as high if high OOP payment is required, or if a fixed sum is covered and patients must bear any excess costs without a clear ceiling (e.g. materials for dentures). Higher requirement to pay OOP for covered healthcare will coincide with more regressivity if utilization of basic healthcare is concentrated among the poor and no options for additional payments are available. However, opportunities and incentives for additional payments, e.g. for additional services, better quality options or more convenience, commonly exist and may provoke higher spending among better-off households. We therefore include the *incentives to pay extra* as a second dimension in which the categories are rated by the degree of choice and the potential price range. Note that we refer to consumer choice only in terms of extra spending, but not in terms of forgoing healthcare: The incentive to pay extra is therefore not related to the decision to pay for healthcare in the first place, but only to the choice to pay for medical goods or services beyond the standard care. We consider incentives to pay for additional healthcare as low when categories have few premium options with little price variability or presumably limited added value, and as high when a wide variety of options with a wide price range is available and increased quality or convenience may be purchased with additional payments. Table 1 gives an overview of the co-payment regulations for the included categories and our assessment of their respective requirement and incentive to pay.

**Table 1:** Co-payments and user charges in the German SHI

Service	Coverage and required co-payments	requirement	incentive
*medicines*			
prescribed medicines	co-insurance of 5-10€ per package (10% up to reference price) + difference between actual and reference price	moderate	moderate
non-prescribed medicines	no coverage	high	moderate
*medical goods and equipment*			
prescribed medical goods	co-insurance of 10%, max. 10€ per month	moderate	moderate
non-prescribed medical goods	no coverage	high	moderate
medical equipment and medical aids	basic options covered with 10% co-insurance, maximum 10€ per month	high	high
*dental care*			
dental care services	free if necessary and effective, additional services not covered	none	moderate
materials for crowns and dentures	partial reimbursement, co-payments can be several hundred Euros even for standard care (*Regelversorgung*), hardship provisions for poor households can be granted	high	high
*ambulatory care*			
ambulatory medical services	free if deemed necessary and effective (then fully covered by SHI)	none	moderate
admission fee	user charge of 10€ per 3 months from 2004–2012	moderate	none
referred services	free if prescribed, no coverage otherwise	none	moderate
*inpatient care*	daily fee of 10€ for a maximum 28 days per year, additional services (e.g. single bed rooms) not covered	moderate	moderate

Coverage, requirement to pay for necessary and effective (here: covered) healthcare and incentive to pay extra in the German SHI, assessment of requirement and incentive to pay by the authors. Spending categories correspond to those included in the EVS data

**Fig. 1:**
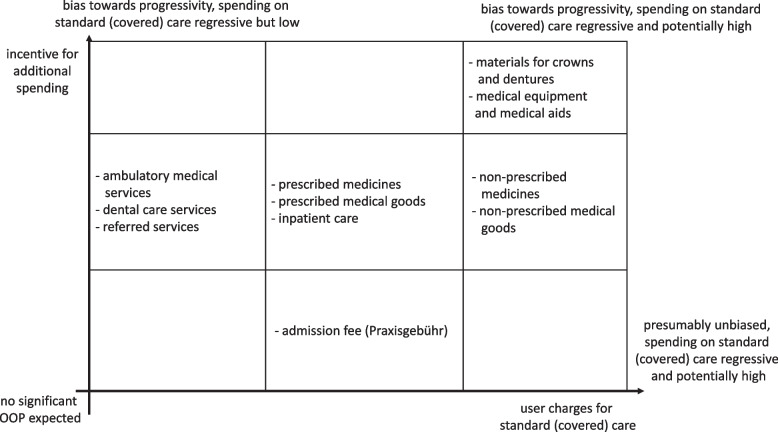
Matrix for rating categories by requirement and incentive to pay. Matrix for rating of categories by requirement to pay for necessary and effective (here: covered) healthcare and by incentive for additional payment for pricier options or additional healthcare, based on authors’ assessment of the co-payment and co-insurance regulations in the German SHI

We combine the two dimensions in Fig. 1 and assign the *requirement to pay OOP for covered care* to the horizontal axis and the *incentive to pay extra* to the vertical axis. The best way to approach Fig. 1 is to consider the different corners: Categories in the bottom-left corner require low OOP spending and will exhibit regressive effects if utilization is concentrated among the poor, but will contribute only little to the overall progressivity or regressivity of OOP spending. Categories in the bottom-right corner will exhibit noticeable OOP spending, which, unless need is concentrated among the rich, can also be expected to be regressive. Categories in the top-left corner will exhibit progressive OOP spending patterns: The presumably small fraction of OOP payments spent for basic options might be regressive if measured separately, but this may be outweighed by higher spending on pricier options or additional healthcare among better-off households. Proportional or progressive distributions in the top-left-corner categories may then point towards consumer choices, if need is not concentrated among the rich. Finally, the top-right corner represents categories where high OOP expenditures required for basic options would yield regressive spending patterns and contribute strongly to an overall regressive effect of total OOP spending. Again, this may be outweighed by higher spending among better-off households for pricier options, such that progressive spending in top-right corner categories may also be interpreted as a hint towards a consumer choices, as long as need is not concentrated among the rich. Note that more consumer choice driven excess spending among the rich is needed to outweigh otherwise regressive spending patterns on covered healthcare in top-right corner categories than in top-left corner categories.

We use the regulations in Table 1 to allocate the spending categories in Fig. 1. For example, we assign prescribed medicines to the center because they have a moderate requirement to pay and a moderate incentive to pay extra. In contrast, we placed dentures and materials in the top-right corner because they have a high requirement to pay even for covered healthcare, and the wide range of quality options involves a considerable incentive to pay more. The distortionary potentials of these two categories are fairly different: OOP spending for prescribed medicines will be regressive when assuming that medical need is not concentrated among the rich [15, 16, 19–22] and that few households pay extra, e.g. to obtain specific brands. For dentures and materials, one would expect a regressive effect for the basic options if need is not concentrated among the rich. However, the incentives for additional spending may bias progressivity measures, as richer households may be more willing and able to choose pricier options.

## Methods

### Data

The EVS [23–25] is conducted by the German Federal Statistical Office every five years and comprises appx. 40,000 households per sample. Each quarter, 25% of the sample households record their revenues and expenditures simultaneously for three months to avoid seasonal effects. See [29] for detailed information on the EVS.

The data for our analysis cover the period from 2008 to 2018 in three waves, which allows us to probe the robustness of our results across time. Our raw samples comprise 44,088 households in 2008, 42,792 households in 2013 and 42,226 households in 2018. We remove households with negative income, assuming reporting errors. We restrict our analysis to households where all members are insured through the SHI for two reasons: First, privately insured individuals are billed by their healthcare providers, reimbursements can be requested from the insurer for up to three years and only pooled reimbursements from all types of private insurers are reported. Linking healthcare spending to reimbursements is therefore impossible and the amounts eventually borne by the households cannot be observed. Second, pooling the public and private insurance sector would yield flawed results as their co-payment schemes have virtually nothing in common. We therefore remove all households with at least one privately insured household member (9,476 households in 2008, 8,888 households in 2013 and 8,912 households in 2018). The final samples comprise 34,529 households in 2008, 33,891 households in 2013, and 33,287 households in 2018.

### Concentration curves and concentration indexes

The concentration curve illustrates the cumulative distribution of some non-negative outcome with respect to income [1, 2, 30, 31], where the cumulative share of the outcome variable of interest *y* is plotted against the cumulative share of the households ranked by income in ascending order. The curve is below (above) the $45^\circ$-line of equality if the outcome is concentrated among the better-off (worse-off). The more the curve deviates from the $45^\circ$-line, the more inequality is observed. The concentration curve is the Lorenz curve if households are sorted by the outcome variable *y*.

The concentration index *C* measures twice the area between the concentration curve and the line of equality [2, 30–32]. We compute *C* as

1 $$\begin{aligned} C=\frac{2}{n\bar{y}}\sum \limits _{i} y_ir_i - 2\bar{r} = \frac{2}{\bar{y}}\text {cov}(y_i,r_i), \end{aligned}$$

where $\bar{y}$ denotes the mean *y* and *n* is the sample size. The weighted fractional rank of the *i*-th household is $r_i=\left( \sum \nolimits _{i=1}^n w_i\right) ^{-1}\left[\left( \sum \nolimits _{j=1}^i w_j \right)- \frac{w_i}{2}\right]$ [4, 22, 32, 33]. *C* is positive (negative), if *y* is concentrated among the better-off (worse-off) and equals zero if no concentration is observed. Here, *C* is the Gini-index if $y=\text {income}$.

### Kakwani’s measure of proportionality

The Kakwani-index $K_\text {OOP}$ for OOP spending on healthcare measures twice the area between the Lorenz curve and the concentration curve. It can be computed as

2 $$\begin{aligned} K_\text {OOP}=C_\text {OOP}-G, \end{aligned}$$

where $C_\text {OOP}$ is the concentration index of OOP spending on healthcare and *G* denotes the Gini index of income. $K_\text {OOP}$ is positive (negative) and indicates progressivity (regressivity) if payments are more (less) concentrated among the better-off than income, i.e. if $C_\text {OOP}>G$ ($C_\text {OOP}< G$) (see also [1–4]). *K* is bounded in a $(-2;1)$-interval where the boundaries represent two extreme hypothetical cases: $K=-2$ indicates that all income is concentrated among the richest and all payments are made by the poorest household, whereas $K=1$ indicates that all households have an equal income, but all payments are made by one single household which is arbitrarily considered as the richest (in fact, $C_\text {OOP}$ and thus also $K_\text {OOP}$ are undetermined in a $(-1;1)$ interval if $G=0$).

### Decomposition of Kakwani’s measure of proportionality

The concentration index of a sum equals the sum of the concentration indexes of its components, weighted by their respective fractions of the total sum [3, 4, 34, 35]. Denoting $x_s$ as the healthcare spending in category $s=1,...,S$ with mean $\bar{x}_s$, and total OOP spending $y=\sum \nolimits _{s=1}^S x_s$ with $\bar{y} = \sum \nolimits _{s=1}^S \bar{x}_s$, Eq. (1) becomes

3 $$\begin{aligned} C_\text {OOP} = \frac{2}{n\bar{y}}\sum \limits _{i} y_i r_i - 1 = \frac{2}{n\bar{y}}\sum \limits _{s} \left( \sum \limits _{i} x_{si}r_i\right) - 1 = \sum \limits _{s} \frac{\bar{x}_s}{\bar{y}} C_s . \end{aligned}$$

The components $\frac{\bar{x}_s}{\bar{y}}C_s$ correspond to the contributions of expenditure category *s* to the inequality in overall spending on healthcare and measure how the overall concentration index would differ if spending in category *s* was equally distributed across incomes with $C_s=0$ [20, 33–35]. Inserting Eq. (3) into Eq. (2) yields

4 $$\begin{aligned} K_\text {OOP} &= C_\text {OOP} - G = \left[ \sum \limits _{s} \frac{\bar{x}_s}{\bar{y}} C_s \right] - G\\ &= \sum \limits _{s} \frac{\bar{x}_s}{\bar{y}} \left( C_s - G \right) = \sum \limits _{s} \frac{\bar{x}_s}{\bar{y}} K_s, \end{aligned}$$

such that the Kakwani index $K_\text {OOP}$ of OOP spending is the sum of the Kakwani indexes $K_s$ of the spending categories weighted by the categories’ respective fraction of total OOP spending. Hence, the term $\frac{\bar{x}_s}{\bar{y}} K_s$ indicates the progressivity of overall OOP spending attributable to spending category *s*: If $K_s>0$ and thus progressive ($K_s< 0$ and thus regressive), then spending category *s* contributes progressivity (regressivity) to the overall effect of OOP (see [3, 4, 7, 36, 37]). The larger the share that $x_s$ has in overall OOP spending, the more relevant is the measured progressivity $K_s$ of category *s* for overall progressivity. We compute the relative contribution of spending category *s* to the overall OOP payments as $\frac{\bar{x}_s}{\bar{y}}\frac{K_s}{K_\text {OOP}}$.

### Computation and statistical inference

All income and expenditure data are observed at the household level and inflated to 2018 Euros. We adjust net income and expenditures using the modified OECD equivalence scale to account for household size and potential economies of scale. The scale assigns a weight of 1 to the first household member, 0.5 to each additional adult member aged 14 or older and 0.3 to each child younger than 14. Net equivalent household income is used for ranking in all computations. We apply sample weights in all computations and assess the accuracy of the estimated concentration indexes, Gini indixes and Kakwani indexes using heteroscedasticity- and autocorrelation-consistent standard errors and Rao’s $\delta$-method [33, 38].

## Results

### Households’ OOP spending on health

Table 2 demonstrates that net incomes increased between 2008 and 2018. OOP spending on healthcare decreased between 2008 and 2013 when the quarterly admission fee was abolished and increased again between 2013 and 2018. OOP spending on healthcare was mostly on non-prescribed medicines, medical equipment and dental care. The changes in the specific categories between the years suggest no clear pattern.

**Table 2:** Monthly income and OOP spending in Euros per equivalent person

	2008	2013	2018
net equivalent household income	1782.24	1823.28	2039.40
total OOP spending on health	44.99	41.73	44.34
*medicines*			
prescribed medicines	4.39	4.82	4.73
non-prescribed medicines	7.74	7.49	7.59
*medical goods and equipment*			
prescribed medical goods	1.08	0.86	1.17
non-prescribed medical goods	2.78	2.49	2.92
medical equipment and medical aids	7.40	6.94	8.02
*dental care*			
dental care services	6.28	7.37	8.04
materials for crowns and dentures	4.80	5.08	4.93
*ambulatory care*			
ambulatory medical services	2.87	2.67	2.82
admission fee	3.21		
referred services	2.29	2.27	2.73
*inpatient care*	2.16	1.74	1.37

Average income and OOP spending on health per equivalent person per month, inflated to 2018 Euros

Spending patterns are mostly in line with the scheme in Fig. 1. Prescribed medicines, ambulatory medical services and referred ambulatory services require low OOP payments and involve moderate incentives for extra payments, and indeed exhibit comparatively low average spending. Medical equipment and aids require high OOP payments and offer high incentives for extra payments in Fig. 1, and Table 2 indicates fairly high OOP spending on them in all years. Despite the required high co-payment and the strong incentives for extra spending for dentures and materials, OOP spending on them is comparatively low. This may be explained by the low fraction of households of $\approx 5\%$ which report spending in this category. Note that those households reported on average payments over 80 Euros per equivalent person. A similar explanation may apply to the low spending on inpatient care, where only around 5% of the households reported payments^1^.

### Redistributive effects of OOP spending on healthcare by categories

The Kakwani-indexes in Table 3 indicate statistically significant regressivity of total OOP spending with the most negative Kakwani index, and thus the strongest regressive effect in 2018. Categories with moderate requirements to pay for covered services and moderate incentives to pay extra, especially prescribed medicines and prescribed medical goods, exhibit strong and statistically significant regressive effects. Medicines and prescribed medical goods in all years and the quarterly admission fee in 2008 are significantly regressive at the 99% level. OOP spending on medical equipment and medical aids and on referred ambulatory services was significantly regressive at the 99% level only in 2018, OOP spending on dental care services was significantly regressive at the 95% level in 2018. In contrast, ambulatory medical services were significantly progressive in 2008 and 2013 at the 99% level. The underlying concentration indexes can be found in Table 4.

**Table 3:** Kakwani indexes for OOP spending on health

	2008	2013	2018
total OOP spending on health	– 0.0581$$^{**}$$	– 0.0494$$^{**}$$	– 0.0805$$^{**}$$
*medicines*			
prescribed medicines	– 0.1385$$^{**}$$	– 0.1674$$^{**}$$	– 0.2094$$^{**}$$
non-prescribed medicines	– 0.1343$$^{**}$$	– 0.1380$$^{**}$$	– 0.1665$$^{**}$$
*medical goods and equipment*			
prescribed medical goods	– 0.1764$$^{**}$$	– 0.1558$$^{**}$$	– 0.1110
non-prescribed medical goods	– 0.1144$$^{**}$$	– 0.1291$$^{**}$$	– 0.1144$$^{**}$$
medical equipment and medical aids	– 0.0127	– 0.0087	– 0.0426$$^{**}$$
*dental care*			
dental care services	– 0.0099	0.0185	– 0.0466$^{*}$
materials for crowns and dentures	– 0.0361	– 0.0276	– 0.0001
*ambulatory care*			
ambulatory medical services	0.1188$$^{**}$$	0.1075$$^{**}$$	0.0383
admission fee	– 0.1994$$^{**}$$		
referred services	– 0.0135	0.0292	– 0.0616$$^{**}$$
*inpatient care*	0.0933	– 0.0299	– 0.0545

Kakwani-indexes of OOP spending on healthcare per equivalent person with respect to net equivalent household income. Negative indexes indicate regressive effects, positive indexes indicate progressive effects

*p<0.05; **p<0.01

**Table 4:** Gini index of income and concentration indexes of OOP spending on health

	2008	2013	2018
net equivalent household income	0.2709$$^{**}$$	0.2728$$^{**}$$	0.2827$$^{**}$$
total OOP spending on health	0.2128$$^{**}$$	0.2235$$^{**}$$	0.2021$$^{**}$$
*medicines*			
prescribed medicines	0.1324$$^{**}$$	0.1054$$^{**}$$	0.0732$$^{**}$$
non-prescribed medicines	0.1366$$^{**}$$	0.1348$$^{**}$$	0.1161$$^{**}$$
*medical goods and equipment*			
prescribed medical goods	0.0944$$^{**}$$	0.1171$$^{**}$$	0.1716$$^{**}$$
non-prescribed medical goods	0.1558$$^{**}$$	0.1438$$^{**}$$	0.1683$$^{**}$$
medical equipment and medical aids	0.2582$$^{**}$$	0.2641$$^{**}$$	0.2401$$^{**}$$
*dental care*			
dental care services	0.2610$$^{**}$$	0.2914$$^{**}$$	0.2361$$^{**}$$
materials for crowns and dentures	0.2348$$^{**}$$	0.2452$$^{**}$$	0.2825$$^{**}$$
*ambulatory care*			
ambulatory medical services	0.3897$$^{**}$$	0.3803$$^{**}$$	0.3210$$^{**}$$
admission fee	0.0715$$^{**}$$		
referred services	0.2574$$^{**}$$	0.3021$$^{**}$$	0.2210$$^{**}$$
*inpatient care*	0.3642$$^{**}$$	0.2430$$^{**}$$	0.2281$$^{**}$$

Concentration indexes (standard errors in parentheses) with respect to net equivalent household income. All indexes are highly statistically significant $(p<0.001)$

*p<0.05; **p<0.01

### Decomposition of the overall regressivity

The most prominent contributors to the overall regressive effects of OOP spending on healthcare in Table 5 are medicines and medical goods, where the non-prescribed fractions contribute more than their prescribed counterparts. The additivity of OOP spending on healthcare allows subtracting the added regressivity of the ambulatory medical services category from the overall regressivity of OOP spending on healthcare in Table 5. Doing so suggests that the regressivity of total OOP spending on healthcare would be appx. 13% higher in 2008, appx. 14% higher in 2013 and appx. 3% higher in 2018 without spending on ambulatory medical services. The counter-example for ambulatory medical services is the quarterly admission fee, which contributed appx. 25% of the observed regressivity of total OOP spending on health.

**Table 5:** Contributions of spending categories to the overall Kakwani indexes for OOP spending on health

	2008		2013		2018	
	contrib	as %	contrib	as %	contrib	as %
total OOP spending on health	– 0.0581	100	– 0.0494	100	– 0.0805	100
*medicines*						
prescribed medicines	– 0.0135	23.27	– 0.0193	39.16	– 0.0223	27.75
non-prescribed medicines	– 0.0231	39.75	– 0.0248	50.19	– 0.0285	35.89
*medical goods and equipment*						
prescribed medical goods	– 0.0042	7.29	– 0.0032	6.52	– 0.0029	3.64
non-prescribed medical goods	– 0.0071	12.24	– 0.0077	15.63	– 0.0075	9.37
medical equipment and medical aids	– 0.0021	3.59	– 0.0014	2.93	– 0.0077	9.56
*dental care*						
dental care services	– 0.0014	2.38	0.0033	– 6.62	– 0.0084	10.49
materials for crowns and dentures	– 0.0038	6.62	– 0.0034	6.82	– 0.0001	0.07
*ambulatory care*						
ambulatory medical services	0.0076	– 13.06	0.0069	– 13.92	0.0024	– 3.03
admission fee	– 0.0142	24.47				
referred services	– 0.0007	1.18	0.0016	– 3.22	– 0.0038	4.72
*inpatient care*	0.0045	– 7.71	– 0.0012	2.52	– 0.0017	2.10

Decomposition of Kakwani-indexes. Negative contributions add regressivity, positive contributions add progressivity. Note that percentage contributions refer to negative overall indexes: positive signs indicate negative contributions, i.e. in the direction of the overall index, and vice versa

## Discussion

This paper developed a framework to assess the potential for consumer choices in different categories of healthcare spending. The first step analyzed user charge policies and distinguished categories by the required OOP spending on basic healthcare and by the potential incentives to pay extra for pricier options. The empirical results support this approach and show that categories with high user charges for basic healthcare contribute regressivity, and that categories with strong incentives for extra payment add no considerable regressivity to the distribution of OOP spending.

On average, better-off households spent lower fractions of their net incomes than worse-off households, and total OOP spending on healthcare was regressive in all years. Nevertheless, all OOP spending on healthcare was significantly concentrated among better-off households (Table 4). Since need is concentrated among poorer households in Germany [15, 20, 39–41] and a substantially different distribution of health in the EVS is unlikely, the results may suggest higher spending on pricier options rather than more utilization of basic healthcare among the better-off.

The results for ambulatory medical services support the notion of consumer choice driven spending on healthcare developed in this paper and demonstrate the effects it may have on progressivity measures. Ambulatory medical services are free of charge if considered necessary and effective. However, $10-12\%$ of the households report spending on ambulatory care in each wave of the EVS, and the statistically significant Kakwani indexes suggest that better-off households spent higher fractions of their incomes than worse-off households. In light of the user charge regulations, spending on ambulatory care can only occur for additional services (*IGeL-Leistungen*), which have not been deemed necessary and effective.

Overall spending on medicines yielded the strongest regressive effect in our analysis in all years, where more regressivity is observed for non-prescribed than for prescribed medicines. Both involve only moderate incentives to pay for pricier options, but co-payments for basic options are capped only for prescribed medicines. Consumer choices may partly explain the weaker regressive effect of non-prescribed medicines, but it remains unclear whether the results reflect forgone optional spending or unmet need. Previous results from Portugal [13] and Austria [12] support the strong regressivity of OOP spending on medicines. Sanwald & Theurl [12] also found prescribed medicines to be more regressive than non-prescribed medicines and support the notion that consumer choices may explain these findings to some extent. In contrast, the observed proportionality for dental care services in 2008 and 2013 and materials for crowns and dentures in all years, which implies a concentration of spending among richer households, may indicate that the better-off opted for higher quality materials and treatments. This interpretation is supported by the mild regressivity of OOP spending on dental care services in contrast to the proportional OOP spending on crowns and dentures.

The Kakwani indexes for spending on inpatient care were small and insignificant. Two explanations may simultaneously apply here: First, the better-off may predominantly pay for options such as single or double rooms, which may cancel out higher spending on basic co-payments by poorer households with higher need. Second, the overall cap on co-payments of 2% of households’ gross income decreases to 1% for the chronically ill [26, 27], which may explain the non-significance of the regressive effect, as maximum payments required from older and poorer individuals will be lower than those required from younger and richer individuals.

Forgone healthcare among the poor may be an alternative explanation for low regressivity or even progressivity in OOP spending on health. User fees may pose considerable access barriers to healthcare, which may lead to unmet healthcare needs among the poor [14, 42, 43]. However, unmet need is unlikely to fully explain these results, because the German healthcare system creates an environment which is close to universal health coverage. Affordable standard options for everything except non-prescribed medicines and medical goods exist (*Regelversorgung*), and exemptions from payment are granted in cases of hardship. As a result, the incidence of unmet need in Germany is rather low: EU-SILC data [44] suggest incidences of unmet need between 0.1-4.1% for medical care and 0.4-10.5% for dental care among individuals aged 16 or older between 2008 and 2019, which declined steadily between 2014 and 2019. This may partially explain the increases in regressivity observed between 2013 and 2018, but not the high levels of progressivity observed for ambulatory medical services, or the low levels of regressivity for dental care services and materials.

In summary, the analysis of progressivity and the decomposition of the Kakwani indexes to measure the contributions of different spending categories to the overall distribution of OOP healthcare spending remains a purely descriptive and non-normative analytical approach, which can be easily applied to other countries with different healthcare systems and attitudes towards economic inequality. The contribution of this paper is the twist in the policy analysis to allow a more nuanced interpretation of this type of results. In order to adapt this to other countries, a profound knowledge of the respective user charge regulations is required to understand and describe the potential for consumer choices among users of healthcare. A challenging exercise will be to adapt the consumer choice bias matrix to settings where the standard care option involves actually inferior quality, or excludes relevant diagnoses or treatments.

This paper has three major limitations. The first is the assumption that decisions on coverage are mainly guided by medical considerations. The SGB V demands that all necessary and effective healthcare must be covered by the SHI, and the decision-making procedures described e.g. in [26, 27] are considered to produce evidence-based results. While exceptions may exist, the low to moderate incidence of unmet need further suggests systematic exclusions of necessary healthcare to be unlikely. The second major limitation is that empirical evidence on incentives to pay extra for pricier options or additional healthcare perceived by patients is, to our knowledge, not available. Consequently, the rating of the incentive to pay extra in different categories had to be mainly based on the authors’ assessments. Although our rating is supported by the results, we encourage further research on patients’ perceptions of different price and quality options in the 11 categories and their income-related willingness to pay for it. The third limitation is that we could not include the importance of different types of services. Especially when thinking about the potential burden of OOP healthcare spending for households, not only the amount to be paid for standard care, but also the urgency and potential consequences of forgoing treatment may be relevant drivers of spending decisions. Including this could either be done by changing the dimensions of the 2-dimensional matrix, or by turning it into a 3-dimensional cube to add the urgency of different services as another dimension. We refrained from this for two reasons: First, the German SHI uses a rather generous concept of necessary and effective care. Benefits go far beyond emergency care and include treatments of chronic and non life-threatening conditions and preventive care, and hardship provisions exist for the poor, which is reflected by the comparatively low incidence of unmet need. Second, and more importantly, the different spending categories cover very heterogeneous treatments. For example, inpatient care includes anything from emergency heart surgery to elective care such as hip replacement, which is all part of the standard care covered by the SHI.

## Concluding remarks

While the definition of a basic healthcare basket is feasible, a major problem is that consumer choices may include extra spending on both higher quality options and superfluous or ineffective healthcare. This would not only mask a part of the regressive effect of OOP spending on basic and necessary healthcare, but may also involve an additional dimension of inequity in the provision of healthcare. Researchers and policymakers should therefore interpret the results for OOP spending in categories with plenty of choice and wide price variations with great caution.

For the German SHI, the results indicate that user charges in categories with limited potential for consumer choice or which are likely to be driven by need add regressivity to the overall distribution of OOP payments. When aiming at a proportional distribution of co-payments, a more effective and more accessible exemption from OOP payments for the poor may help to reduce the over-proportional financial burden among them. Alternatively, a reduction of user charges in general might also further reduce the incidence of unmet need, but policymakers should always weigh the benefits of reducing financial access barriers against potential disincentives for over-utilization when changing user charges.

## Data Availability

The data that support the findings of this study are available from the Research Data Centers of the Federal Statistical Office and Statistical Offices of the Länder in Germany but restrictions apply to the availability of these data, which were used under license for the current study, and so are not publicly available. See [23–25] for details.

## Abbreviations

OOP: Out-of-pocket
SHI: Statutory Health Insurance
EVS: Einkommens- und Verbrauchsstichprobe
SGB: Social Code Book (Sozialgesetzbuch)

## Footnotes

1 These figures indicate the fraction of households reporting any spending within a given quarter year and are not necessarily comparable to yearly incidence rates.
